# Anastrozole and levonorgrestrel-releasing intrauterine device in the treatment of endometriosis: a randomized clinical trial

**DOI:** 10.1186/s12905-021-01347-9

**Published:** 2021-05-20

**Authors:** Pedro Acién, Irene Velasco, Maribel Acién

**Affiliations:** 1 Department/Division of Gynecology, Miguel Hernández University, San Juan Campus, 03550 San Juan, Alicante, Spain; 2 Obstetrics and Gynecology Service, San Juan University Hospital, 03550 San Juan, Alicante, Spain; 3 Departamento/Area de Ginecología, Facultad de Medicina de La Universidad “Miguel Hernández”, Campus de San Juan, 03550 Alicante, Spain

**Keywords:** Aromatase inhibitors, Anastrozole, Levonorgestrel-IUD, Endometriosis, Endometriomas, Clinical trial

## Abstract

**Background:**

To study the effectiveness of an aromatase inhibitor (Anastrozole) associated with levonorgestrel-releasing intrauterine device (LNG-IUD, Mirena®) in the treatment of endometriosis.

**Methods:**

Prospective, randomized clinical trial. Setting: University Hospital (single center). Elegibility criteria: Endometriomas > 3 × 4 cm, CA-125 > 35 U/mL and endometriosis symptoms. Patients: Thirty-one women randomized to anastrozole + Mirena® + Conservative Surgery(CS) (n = 8), anastrozole + Mirena® + transvaginal ultrasound-guided puncture-aspiration (TUGPA) (n = 7), Mirena® + CS (n = 9), or Mirena® + TUGPA (n = 7). Interventions: Anastrozole 1 mg/day and/or only Mirena® for 6 months; CS (ovarian and fertility-sparing) or TUGPA of endometriomas one month after starting medical treatment. Main Outcome Measures: Visual analogic scale for symptoms, CA-125 levels, ultrasound findings of endometriomas and recurrences.

**Results:**

A significant improvement in symptoms during the treatment (difference of 43%, 95% CI 29.9–56.2) occurred, which was maintained at 1 and 2 years. It was more significant in patients including anastrozole in their treatment (51%, 95% CI 33.3–68.7). For CA-125, the most significant decrease was observed in patients not taking anastrozole (73.8%, 95% CI 64.2–83.4 vs. 53.8%, 95% CI 25.7–81.6 under Mirena® + anastrozole). After CS for endometriosis, a reduction of ultrasound findings of endometriomas and long-term recurrence occurred, with or without anastrozole. At 4.2 ± 1.7 years (95% CI 3.57–4.85), 88% of the patients who underwent CS were asymptomatic, without medication or reoperation, compared to only 21% if TUGPA was performed, with or without anastrozole (*p* = 0.019).

**Conclusions:**

Dosing anastrozole for 6 months, starting one month before CS of endometriosis, reduces significantly the painful symptoms and delays recurrence, but has no other significant advantages over the single insertion of LNG-IUD (Mirena®) during the same time. Anastrozole and/or only Mirena® associated with TUGPA are not effective.

***Trial registration*:**

Eudra CT System of the European Medicines Agency (London, 29-Sept-2008) Nº EudraCT: 2008-005744-17 (07/11/2008). Date of enrolment of first patient: 15/01/2009.

**Supplementary Information:**

The online version contains supplementary material available at 10.1186/s12905-021-01347-9.

## Background

The therapeutic perspectives for endometriosis have been directed in the last years towards the use of a variety of new medications including immunomodulatory agents (i.e. local interleukin-2r [1]), selective estrogen and progesterone receptor modulators, GnRH antagonist, angiogenic inhibitors, or the third-generation aromatase inhibitors (AI) (i.e. Anastrozole and Letrozole), systemically and/or locally administered, and eventually without the need for surgery [2–5]. Anastrozole inhibits aromatase and decreases the amount of estrogens in all tissues; thus, in positive aromatase endometriotic implants, it should prevent local estrogenic production and, therefore, endometriotic tissue proliferation [2, 6–8]. That estrogen suppression and subsequent response from the adenohypophysis could lead to an increase in gonadotropins with consequent ovarian stimulation and possible formation of dysfunctional cysts or eventual follicular rupture, ovulation, and pregnancy. Thus, in all studies in premenopausal women with endometriosis in which long-term AIs have been used, they have been associated with gonadotropin-releasing hormone (GnRH) analogues, to slow down gonadotropins and produce hypoestronism, or with progestins or oral contraceptive pills (OCP), which could also improve endometriosis and its symptoms. An alternative contraceptive method could be the levonorgestrel-releasing intrauterine device (LNG-IUD), Mirena®, a device that releases 20 μg/24 h of levonorgestrel (LNG) in situ. LNG induces the atrophia of eutopic endometrium that would reduce or avoid the retrograde menstruation. Based on previous reports about the use of both medications (Anastrozole [3, 4] and Mirena® [9]) in endometriosis, our therapeutical proposal was to associate Anastrozole administered orally for 6 months with Mirena® during the same time period. Furthermore, although this medical treatment (Anastrozole and/or only Mirena) could be more effective when associated to the surgical exeresis of the endometriotic foci, we wanted to clarify whether this would also be the case when associated to transvaginal ultrasound-guided puncture-aspiration (TUGPA) of the endometriomas, as we had already done in previous works associating TUGPA with analogues or leaving r-IL2 in situ [1, 10, 11].

### Study objective

We proposed a clinical trial (CT), whose main objective was to assess the efficacy of AI Anastrozole associated with LNG-IUD (Mirena®) (compared with Mirena® alone), in the treatment of moderate and severe endometriosis and its symptoms, along with laparoscopic or laparotomic conservative surgery (CS) (ovarian and fertility-sparing), or with simple TUGPA of the endometriomas.

## Methods

### Study design

Randomized, comparative and controlled clinical trial (CT) whose protocol (ENDOMET-IA-DIULNG08) was approved by the Ethics Committee of San Juan University Hospital (26-August-2008). It was registered in the Eudra CT System of the European Medicines Agency (London, 29-Sept-2008; Nº EudraCT: 2008-005744-17) (07/11/2008) and then authorized by the Spanish Agency for Medicines and Health Products (AEMPS) (10/11/2008).

### Participants

Premenopausal women with endometriomas recruited at the Endometriosis and Reproductive Medicine Consultation of San Juan University Hospital. They had been advised for CS, did not currently desire pregnancy, and accepted the insertion of LNG-IUD (Mirena®) for 6 months and randomization to perform conservative surgery (CS) with ovarian and fertility-sparing by laparoscopy or laparotomy, or only transvaginal ultrasound-guided puncture-aspiration (TUGPA) of endometriomas, 1 month after the IUD insertion.

### Inclusion criteria

Young women (< 41 y) with significant clinical symptoms (score of visual analogue scale (VAS) ≥ 4), elevated CA-125 (≥ 35 U/mL), and a transvaginal ultrasound (TVU) with suggestive findings of endometriomas (cyst > 3 × 4 cm with ground glass echogenicity and no papillary structures with detectable blood flow). These patients could have a previous diagnosis and treatment (medical and/or surgical) of endometriosis, but they should not have received medical treatment in the last 3 months.

### Exclusion criteria

(i) Pregnancy; (ii) Infertility with current desire for pregnancy; (iii) No previous sexual intercourse and/or non-acceptance of insertion of Mirena®; (iv) Acute or recurrent pelvic inflammatory disease or genital tract infection; (v) Uterine malformations and/or leiomyomas; (vi) Any medical pathology that could contraindicate the treatment with Anastrozole or LNG-IUD (Mirena®).

Written informed consent was obtained from all patients before *randomization*. Participants were randomized by computer, determined at the Hospital Pharmacy after a telephone call from the Endometriosis Consultation. Between January 15, 2009 (date of inclusion of the first patient) and March 15, 2015, the eligibility criteria were analyzed in 52 patients who had ovarian cystic tumors suggestive of endometriomas, with indication to CS. After excluding 21 patients due to not meeting all the inclusion criteria, doubts in the ultrasound diagnosis, or because they declined to participate, the other 31 women were included, randomized, treated and followed up according to the following subgroups: (1) Anastrozole-Mirena-CS (n = 8); (2) Anastrozole-Mirena-TUGPA (n = 7); (3) no Anastrozole-Mirena-CS (n = 9); and (4) no Anastrozole-Mirena-TUGPA (n = 7).

### Procedures

Medical treatments: (1) oral Anastrozole, 1 tablet of 1 mg daily for 6 months administered to patients in subgroups 1 and 2; (2) LNG-IUD (Mirena®) for all patients; (3) calcium carbonate and cholecalciferol (Ca + Vitamin D) to patients taking Anastrozole to avoid the accelerated bone loss effect of AI. Surgical treatments: (1) laparoscopy (in 13 women, 76%) or laparotomy (in 4 women—in 2 was conversion—) with CS of endometriosis (cystectomy and adhesiolysis); or (2) TUGPA of endometriomas (in 14 women). All surgeries were performed or directed by the first author of the present study (PA).

### Research plan

All patients will undergo a first analytical control, clinical exploration and TVU in the second half of the cycle, being randomized according to the subgroups previously exposed. Patients of subgroups 1 and 2 would start taking Anastrozole at the beginning of the next menstruation, placing the LNG-IUD (Mirena®) during it, as well as in the other subgroups. At the time, the surgical proposal to be practiced a month later was processed. Postoperative control follow-ups would be done at 3 and 6 months (time of withdrawal of Anastrozole and Mirena®); thereafter at 9, 12, 18, 24 months, and then annual follow-ups.

### Assessments

All patients must have a detailed medical history about their antecedents and previous treatments, also including clinical exploration, TVU, hormonal and tumor marker analysis (CA-125, CA-19–9) and symptoms score using our VAS [maximum 10 points, including dysmenorrhea (0–3), deep dyspareunia (0–3), chronic pelvic pain (CPP, 0–3), and others (0–1)]. In all subsequent follow-ups, TVU, analysis and VAS score for symptoms were repeated.

We considered *recurrence* of the disease when an endometrioma was detected in any control, which persisted or grew in subsequent follow-ups, associated with an increase in VAS score and/or CA-125 level. In any case, the recurrences of small endometriomas (1.5–3 cm) and endometriomas greater than 3 × 4 cm are presented separately in the tables of results.

### Outcomes

#### Primary endpoint

Clinical, analytical and ultrasound improvement assessed by (1) reduction or disappearance of symptoms; (2) normalization of CA-125 values; (3) reduction or disappearance of endometriomas. These parameters were studied in each postoperative control follow-up at 3 and 6 months; thereafter at 9, 12, 18, 24 months, and then in annual follow-ups. *Secondary endpoints*: (1) decrease or disappearance of recurrences; (2) rate of reoperations; (3) subsequent pregnancy achievement; and (4) valuation of the clinical state in the last follow-up and the need for other treatments.

### Safety and adverse events

In laparoscopic or laparotomic surgery, peritoneal fluid and biopsies or surgical specimens were collected for cytological and histopathological studies. In TUGPA, endometrioma fluids were also collected for cytological analysis. Side effects and adverse events were registered and considered in each patient follow-up.

### Statistical analysis

#### Sample size

Based on previously published studies using AI [3, 4, 12, 13] or LNG-IUD [9], in the research project-protocol of the clinical trial ENDOMET-IA-DIULNG08 (EudraCT No.: 2008-005-744-17) we include the following analysis: “with respect to symptoms, with the LNG-IUD we expect to find improvements of 60% and, with associating aromatase inhibitors + LNG-IUD, of 94% (*p* < 0.05 with 16 cases in each branch). Regarding recurrence of symptoms and endometriosis, after LNG-IUD it would be foreseeable that only 20–25% will be free of recurrence in two years, while with aromatase inhibitors + LNG-IUD, more than 60% may be free (*p* < 0.01 with 24 cases in each branch)”. Therefore, we estimated a total sample size of 48 patients (12 in each subgroup) to study in a period of 3 years, which was prolonged another 3 years due to difficulties for recruitment. However, we decided to finish it in March 2015 due to its low rate, to the need to make a final report in September-2015 and also because the patients undergoing TUGPA were not showing good clinical results. Nevertheless, their follow-up continued to the final data collection in July-2017.

All data were entered into SPSS Statistics version 25.0 (IBM, Spain) to perform statistical analysis. Data are expressed as percentages, mean ± standard deviation (SD), median, minimum and maximum (min–max) values, and a 95% confidence interval (CI) (if applicable). The main dependent variables were the VAS score, the absence or presence of endometriomas in TVU, and the values of CA-125, as well as their evolution in later follow-ups. The main independent variables were Anastrozole + LNG-IUD or only LNG-IUD treatments, as well as CS or TUGPA of endometriomas. We applied descriptive statistical analysis for qualitative variables to determine frequencies and distribution using contingency tables and comparison of proportions. The chi-squared, Kruskal–Wallis, Mann–Whitney U and correlation tests were used to compare groups and parameters in the different follow-ups. For quantitative or numerical variables, we applied nonparametric tests for paired data to compare the values before and at 3, 6, and 9 months, and 1 and 2 years, and the last follow-up after treatment, calculating the Wilcoxon signed rank test, the signs test, the McNemar test (for dichotomous variants), and the marginal homogeneity test, noting in tables and graphs only the significant results. Likewise, nonparametric tests were applied to compare Anastrozole and non-Anastrozole groups. To determine the recurrence and reoperation rates, we calculated the percentage accumulated every 3 months up to 2 years, and then every year up to 6 years, pointing at the corresponding figures the patients at risk in each period, subtracting recurrences and loss to follow-up. All *p* values reported are 2-tailed, and *p* < 0.05 was considered significant.

This study adheres to CONSORT guidelines.

## Results

### Participants recruited and included in the study

**Fig. 1:**
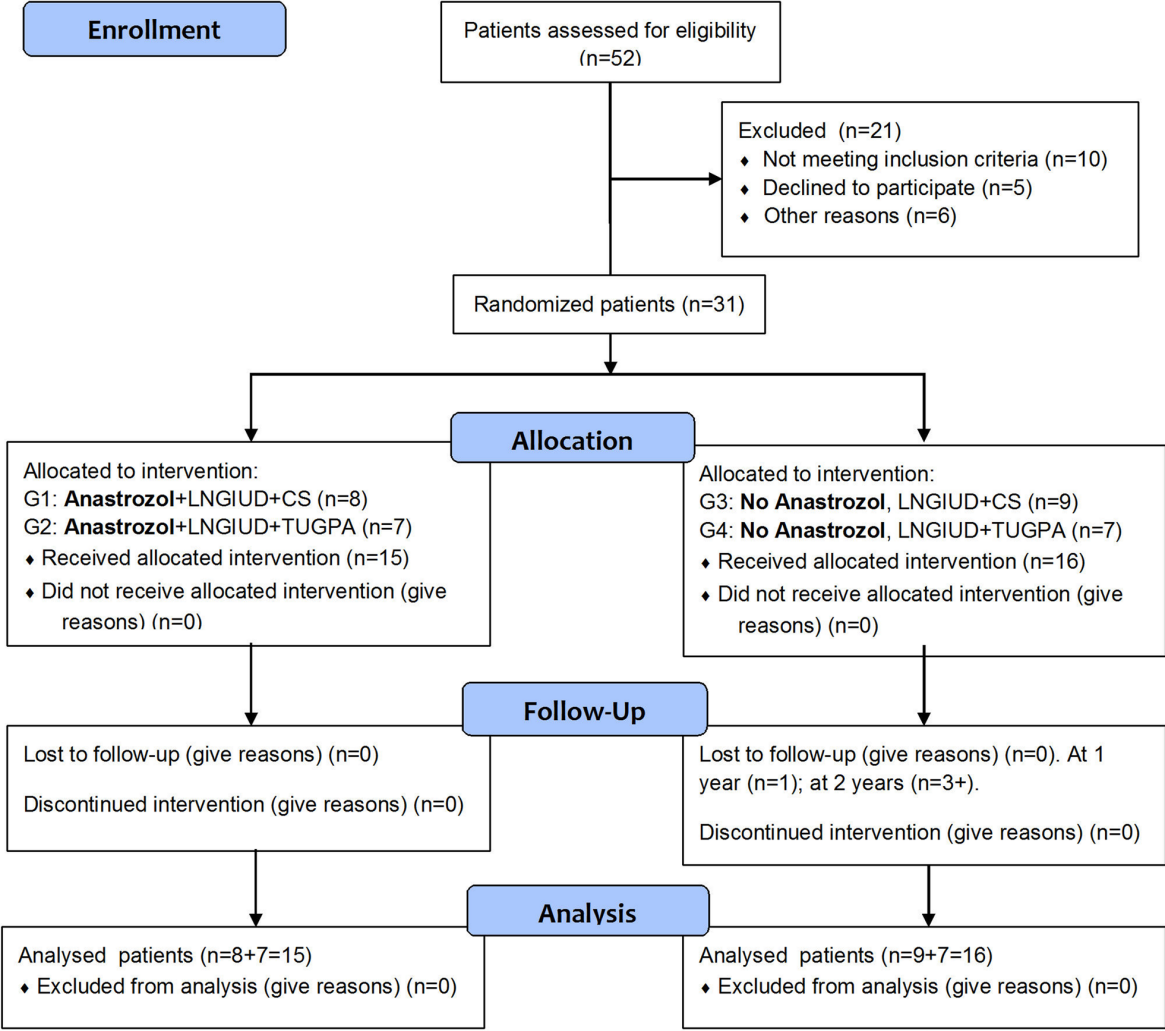
Trial profile

The eligibility criteria were analyzed in 52 patients (see Fig. 1). After excluding 21 patients, 31 women were included in the CT and randomized in the 4 subgroups mentioned above. All patients had LNG-IUD (Mirena®) for 6 months, and 15 of them also received Anastrozole for the same period. The latter were followed for 4.67 ± 1.63 years (95%CI: 3.76–5.57), and the first ones for 3.78 ± 1.77 years (95%CI: 2.84–4.72), without significant differences.

### Baseline characteristics

Table 1 shows the characteristics, antecedents, symptoms, laboratory tests, and ultrasound and operative findings of the four subgroups of patients. No significant differences were observed**.**

**Table 1:** Baseline characteristics of the patients included in the Clinical Trial

Characteristics	G1. Anastrozol + LNGIUD + CS (n = 8)	G2. Anastrozol + LNGIUD + TUGPA (n = 7)	G3. LNGIUD + CS (n = 9)	G4. LNGIUD + TUGPA (n = 7)
Age (y)	30.7 ± 7.3 (21–40)	31.0 ± 5.6 (24–40)	33.6 ± 4.0 (26–40)	30.4 ± 8.2 (20–40)
Parity ≥ 1	3 (37.5%)	1 (14.3%)	5 (55.5%)	0 –
Infertility	0 –	1 (14.3%)	1 (11.1%)	1 (14.3%)
*Antecedents*				
a. Endometriosis, MST	1 (12.5%)	1 (14.3%)	2 (22.2%)	2 (28.6%)
b. Endometriosis + myomas	1 (12.5%)	1 (14.3%)	0 –	0 –
c. Endometriosis-OCP	0 –	1 (14.3%)	0 –	2 (28.6%)
*Symptoms*: VAS/10	5.6 ± 1.6 (3–8)	6.1 ± 2.0 (4–9)	5.6 ± 2.2 (3–9)	4.7 ± 2.1 (3–8)
Dysmenorrhea/3	2.1 ± 0.2 (2–2.5)	2.1 ± 0.6 (1.5–3)	1.7 ± 0.6 (0.5–2)	1.8 ± 0.9 (0.5–2.5)
Dyspareunia/3	(7) 1.1 ± 0.9 (0.5–3)	1.6 ± 1.1 (0–3)	1.4 ± 1.0 (0–3)	(4) 0.9 ± 1.0 (0–2)
CPP/3	1.7 ± 0.9 (0–2.5)	1.4 ± 0.8 (0.5–2.5)	1.7 ± 1.0 (0–3)	1.8 ± 1.5 (0–3)
*Trasvaginal US/ovaries*				
a. Endometriomas, RO	2 (25%)	0 –	4 (44.4%)	0 –
b. Endometriomas LO	2 (25%)	3 (42.8%)	2 (22.2%)	5 (71.4%)
c. Bilateral/kissing ovaries/rvs	4 (50%)	4 (57.1%)	3 (33.3%)	2 (28.6%)
*Analysis*				
CA-125	80.5 ± 57.8 (36–190)	90.4 ± 37.6 (37–150)	56.3 ± 12.9 (36–70.4)	87 ± 42 (53–168)
CA-19-9	44.9 ± 63.5 (9.5–198)	(6)58.9 ± 71 (2–191)	(8)38 ± 19.4 (11–66)	(6)36.6 ± 37 (11–111)
*Diagnosis*				
a. Endometrioma, RO	1 (12.5%)	0 –	3 (33.3%)	0 –
b. Endometrioma, LO	1 (12.5%)	3 (42.8%)	2 (22.2%)	4 (57.1%)
c. Pelvic endometriomas	6 (75%)	4 (57.1%)	3 (33.3%)	3 (42.8%)
d. Recurrent endomet/rvs	0 –	0 –	1 (11.1%)	0 –
*Surgery*				
a. TUGPA	0 –	7 (100)	0 –	7 (100)
b. Laparoscopy, CS	7 (87.5%)	0 –	6 (66.7%)	0 –
c. Laparotomy, CS	1 (12.5%)	0 –	3 (33.3%)	0 –
*Findings in CS*				
a. Endometriomas	4 (50%)	–	5 (55.5%)	–
b. Severe pelvic endomet + end-omas	3 (37.5%)	–	4 (44.4%)	–
c. Endometriomas + myoma	1 (12.5%)	–	0 –	–
*Histopathology*				
a. Cytology compatible with endometriosis	–	7 (100)	–	7 (100)
b. Endometriosis (cystic)	8 (100) (1 + Myo)	0 –	7 (77.7%)	0 –
c. Atypical endometriosis	0 –	0 –	2 (22.2%)	0 –

Data are n(%), mean ± standard desviation (SD) and (min–max) values. TUGPA, transvaginal ultrasound-guided puncture-aspiration; CS, conservative surgery; MST, previous medical and surgical treatment; OCP, oral contraceptive pill; VAS, visual analogic scale; CPP, chronic pelvic pain; RO, right ovary; LO, left ovary; endomet, endometriosis; rvs, recto-vaginal septum

### Primary outcomes

The evolution of the primary efficacy variables, up to 2 years, in the four studied groups is showed in Table 2. In the Anastrozole groups, especially in + CS, the VAS values (mainly dysmenorrhea) were significantly reduced and kept low. Similar results were observed in the levels of tumor markers and the recurrence of endometriomas, but differences were not significant if + TUGPA was performed. The reduction of VAS (dysmenorrhea and dyspareunia), CA-125, and endometriomas was equally significant in patients treated with LNG-IUD + CS without Anastrozole. CA-125 level also decreased significantly in the LNG-IUD + TUGPA –No Anastrozole group.

**Table 2:** Evolution of the primary efficacy variables in the 4 randomized groups of the Clinical Trial

Group	Variable	Before Treatment (N), m ± SD or n(%)	3 months (DT) (N), m ± SD or n(%)	6 months (DT) (N), m ± SD or n(%)	9 months (3 m AT) (N), m ± SD or n(%)	1 year (6 m AT) (N), m ± SD or n(%)	2 years (1.5y AT) (N), m ± SD or n(%)
**(1) Anastrozol + LNG-IUD**							
** + CS**	*VAS/10*	(8) 5.6 ± 1.6	(8) 2.4 ± 1.2^a1b2d2^	(8) 2.4 ± 2.1^a1d1^	(8) 2.3 ± 1.3^a1b2d2^	(8) 2.6 ± 1.6^a1b2d1^	(8) 2.4 ± 1.6^a1d1^
	Dysmenorrhea	2.1 ± 0.2	1.1 ± 0.7^a1b1d1^	0.6 ± 0.8^a1b1d2^	1 ± 0.5^a2b2d2^	1.2 ± 0.9^a1b1d1^	1.1 ± 0.7^a1b1d1^
	Dyspareunia	(7) 1.1 ± 0.9	(7) 0.4 ± 0.5	(7) 0.6 ± 1.1	(7) 0.4 ± 0.6^a1b1d1^	(7) 0.9 ± 1.8	(7) 0.4 ± 0.7
	CPP	1.8 ± 0.9	0.6 ± 0.5^a1b1d1^	0.6 ± 0.4^a1b1d1^	0.7 ± 0.5^a1b1d1^	0.8 ± 0.5^a1b1d1^	1.2 ± 1.6
	*TV ultras/ovaries*						
	Normal or dysfunctional	–	7 (87.5)	5 (62.5)	6 (75)	7 (87.5)	5 (62.5)
	Simple cyst or small endomet	–	1 (12.5)	3 (37.5)	2 (25)	1 (12.5)	2 (25)
	Endometriomas (rvs)	8 (100)	0–^a2b2d2^	0–^a2b2d2^	0–^a2b2d2^	0–^a2b2d2^	1 (12.5)^a1b1d1^
	*Tumor markers*						
	CA-125	(8) 80.5 ± 57.8	(7) 19.5 ± 10.9^a1d1^	(8)11.3 ± 3.6^a1b2d1^	(7) 24.2 ± 26.2	(7)19.7 ± 10.5^a1b1d1^	(8)19.5 ± 10.5^a1b2d1^
	CA-19-9	(8) 44.9 ± 63.5	(7) 10.9 ± 8.6^a1^	(6) 6 ± 3.1^a1b1^	(6) 17.2 ± 22.1^a1^	(6) 12.7 ± 13.8^b1^	(7) 18.7 ± 26.8
**(2) Anastrozole + LNG-IUD**							
** + TUGPA**	*VAS/10*	(7) 6.1 ± 2	(7) 3.1 ± 3.1^a1d1^	(7) 3.6 ± 2.1^a1b1d1^	(7) 4.9 ± 2.5^a1d1^	(5) 3.6 ± 1.1	(4) 3.1 ± 1
	Dysmenorrhea	2.1 ± 0.6	0.5 ± 0.9^a1b1d1^	0.6 ± 0.6^a1b1d1^	1.4 ± 0.4^a1d1^	1.5 ± 0.8	1.4 ± 1
	Dyspareunia	1.6 ± 1.1	0.9 ± 1	1.1 ± 1.1	1 ± 1	0.5 ± 0.4	0.4 ± 0.2
	CPP	1.4 ± 0.8	0.8 ± 0.9	1.4 ± 1.1	1.5 ± 1	1.1 ± 0.7	0.8 ± 0.5
	*TV ultras/ovaries*					2 op (28.6)	3 op (42.9)
	Normal or dysfunctional	–	1 (14.3)	0–	0–	0–	0–
	Simple cyst or small endomet	–	3 (42.9)	1 (14.3)	1 (14.3)	0–	3 (75) (42.9)
	Endometriomas (rvs)	7 (100)	3 (42.9)	6 (85.7)	6 (85.7)	5 (100) (71.4)	1 (25) (14.3)
	*Tumor markers*						
	CA-125	(7) 90.4 ± 37.6	(7) 69.8 ± 105.7	(7) 74 ± 70.9	(6) 63.9 ± 31.1	(5) 71.1 ± 71.6	(4) 50.6 ± 36.1
	CA-19-9	(6) 58.9 ± 71	(7) 21.3 ± 16.5	(6) 64.9 ± 100.8	(5) 29.8 ± 22.2	(4) 46 ± 56.3	(4) 17.5 ± 11.8
**(3) LNG-IUD, No Anastrozol**							
** + CS**	*VAS/10*	(9) 5.6 ± 2.2	(9) 2.9 ± 2^a1d1^	(9) 3.1 ± 1.8^a1d1^	(9) 3.5 ± 1.9^a1b1d1^	(8) 4 ± 2^a1b1d1^	(6) 2.8 ± 1.1
	Dysmenorrhea	1.7 ± 0.6	0.8 ± 0.7^a1b1d1^	0.8 ± 0.7^a1b2d1^	1.4 ± 0.9	1.5 ± 1	(5) 1.3 ± 1
	Dyspareunia	1.4 ± 1	(8) 0.8 ± 1	(8) 0.7 ± 0.5^a1b1d1^	(8) 0.7 ± 0.7^a1b1d1^	(7) 0.9 ± 0.7^a1d1^	(5) 0.4 ± 0.5
	CPP	1.7 ± 1	0.8 ± 0.9	1 ± 0.7	0.8 ± 0.8	1 ± 1	(5) 0.4 ± 0.5
	*TV ultras/ovaries*						
	Normal or dysfunctional	–	8 (88.9)	8 (88.9)	5 (55.6)	5 (62.5)	3 (50)
	Simple cyst or small endomet	–	0–	1 (11.1)	1 (11.1)	2 (25)	2 (33.3)
	Endometriomas (rvs)	9 (100)	1 (11.1)	0–	2 (22.2)	1 (12.5)	1 (16.7)
	*Tumor markers*						
	CA-125	(9) 56.3 ± 12.9	(8) 11.4 ± 7.9 ^a1b2^	(9)12.4 ± 8.9^a2b2d2^	(9) 16 ± 9.1^a2b2d2^	(8)16.7 ± 10.8^a1b2d2^	(6) 21.2 ± 19^a1b1d1^
	CA-19-9	(8) 38 ± 19.4	(6) 10.2 ± 4^a1^	(8) 14.8 ± 11.3^a1d1^	(8) 11.9 ± 6.5^a1b1d1^	(7) 12.1 ± 6.8^a1b1d1^	(6) 25.5 ± 22.2
**(4) LNG-IUD, No Anastrozol**							
** + TUGPA**	*VAS/10*	(7) 4.6 ± 2.2	(7) 3.4 ± 1.8	(7) 3 ± 1.3	(7) 3.2 ± 1.5	(6) 3.6 ± 1.6	(2) 4.3 ± 2.5
	Dysmenorrhea	1.6 ± 0.7	1.4 ± 0.9	1.1 ± 0.9	1.4 ± 0.6	1.3 ± 0.5	(1) 1
	Dyspareunia	(4) 0.9 ± 1	(6) 0.3 ± 0.4	(6) 0.3 ± 0.4	(5) 0.2 ± 0.4	(4) 0.5 ± 1	(1) 2
	CPP	1.6 ± 1.1	1.1 ± 0.9	1.1 ± 0.9^a1d1^	0.9 ± 0.7	1.1 ± 0.9	(1) 2
	*TV ultras/ovaries*					(1 op, 14.3%)	(3 op, 42.9%)
	Normal or dysfunctional	–	1 (14.3)	0–	0–	1 (16.7) (14.3)	1 (50) (14.3)
	Simple cyst or small endomet	–	2 (28.6)	0–	0–	1 (16.7) (14.3)	0–
	Endometriomas (rvs)	7 (100)	4 (57.1)	7 (100)	7 (100)	4 (66.7) (57.1)	1 (50) (14.3)
	*Tumor markers*						
	CA-125	(7) 87 ± 42	(7)26.9 ± 18.1^a1b1d1^	(7)26.6 ± 21.9^a1b1d1^	(7)42.2 ± 37.8^a1b1d1^	(6) 43.2 ± 24.2^a1d1^	(2) 51.7 ± 3.4
	CA-19-9	(6) 36.6 ± 37.4	(5) 15.1 ± 14.3	(6) 12.3 ± 13^a1^	(5) 21.2 ± 22.3	(5) 18.5 ± 13.2	(2) 18.9 ± 13.7

Data are n(%), mean ± standard desviation (SD). Nonparametric tests (for two related samples) between values before treatment and at 3 m, 6 m, 9 m, 1y and 2y.– Test of ranges with Wilcoxon signs: a1 = *p* < 0.05, a2 = *p* < 0.01; a3 = *p* < 0.001. Signs test: b1 = *p* < 0.05, b2 = *p* < 0.01, b3 = *p* < 0.001. Mc Nemar test (for dichotomous variants): c1 = *p* < 0.05, c2 = *p* < 0.01; c3 = *p* < 0.001. Marginal homogeneity test: d1 = *p* < 0.05, d2 = *p* < 0.01, d3 = *p* < 0.001. Only significant results are indicated. DT, during treatment; AT, after treatment; CS, conservative surgery; TUGPA, transvaginal ultrasound-guided puncture-aspiration; VAS, visual analogic scale; CPP, chronic pelvic pain; TV ultras, transvaginal ultrasound; endomet, endometrioma; rvs, rectovaginal septum; op, operated

**Fig. 2:**
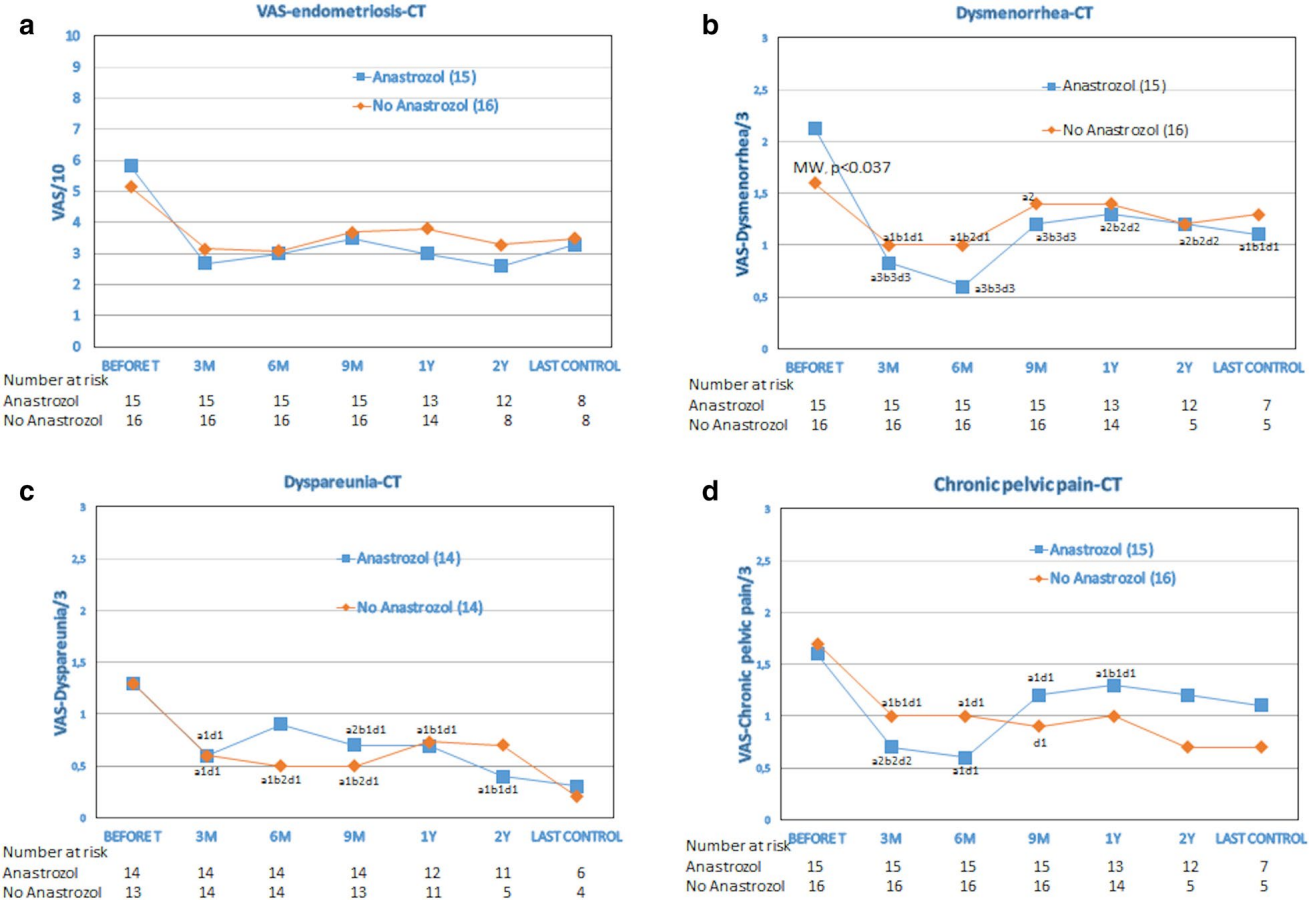
Evolution of the symptoms score in patients taking or not Anastrozole: **a** VAS. **b** Dysmenorrhea. **c** Dyspareunia. **d** Chronic pelvic pain

**Fig. 3:**
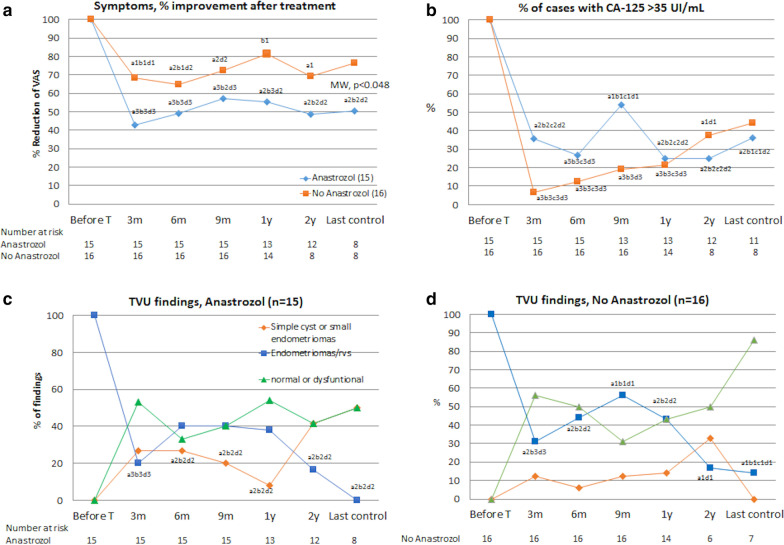
Evolution of the variables of primary efficacy taking or not Anastrozole: **a** VAS, % improvement by treatment. **b** % of cases with CA-125 > 35 UI/mL. **c** Ultrasound finding (TVU) in the CT under Anastrozole (15 patients). **d** Ultrasound finding (TVU) in the CT, no Anastrozole (16 patients). Normal or dysfunctional = findings of normal ovaries or with dysfunctional cyst (luteal)

A more detailed assessment of this evolution is represented in Fig. 2 (and in Fig. 3), according to the patients who took or not Anastrozole (in follow-up results, the re-operated cases were excluded). Figure 2 represents the evolution of the symptoms scaled over 10 (absolute value of the VAS), as well as the evolution of dysmenorrhea, dyspareunia, and CPP scaled over 3. The most significant improvements were for dysmenorrhea and CPP during the taking of Anastrozole. Figure 3a shows that the percentage of reduction or improvement of the VAS score ​​is more significant at 3 and 6 months, with or without Anastrozole, maintaining similar values during the follow-up period. On average, there was a significant improvement of symptoms during (difference 44%, 95%CI: 27.7–60.4) and after treatment at 6 months (43%, 95%CI: 29.9–56.2), which is maintained at follow-up of 1 year (31%, 95%CI: 17.6–44.4) and 2 years (43.7%, 95%CI: 27.7–59.6). This improvement was more significant in those patients who had taken Anastrozole (at 3 months: 57%, 95%CI: 40.3–73.4; at 6 months: 51%, 95%CI: 33.3–68.7; at 1 year: 44.5%, 95%CI: 28–61; and at 2 years: 51.3%, 95%CI: 31.6–71). There are, however, no significant differences between Anastrozole and non-Anastrozole, except for at the 1-year follow-up (Mann–Whitney U test, *p* = 0.048).

Regarding levels of CA-125 (Fig. 3b), we observed significant reduction, similar to that of the VAS score, but only in those patients who did not take Anastrozole, in both the absolute values and the percentage of cases with high level of this marker (> 35 U/mL), at least up to 1 year. In these patients, the decrease of CA-125 values was 64% (95%CI: 40.8–87.3) at 3 months and 73.8% (95%CI: 64.2–83.4) at 6 months versus 49.6% (95%CI: 19.6–79.7) and 53.8% (95%CI: 25.7–81.6), respectively, in patients treated with Anastrozole + Mirena®.

The sonographic findings showed similar behaviour (Fig. 3cd), with a progressive increase of recurrent endometriomas until the 9 or 12 months controls that decreased when some patients were re-operated, but no significant differences were observed between Anastrozole and non-Anastrozole groups.

### Secondary outcomes

**Fig. 4:**
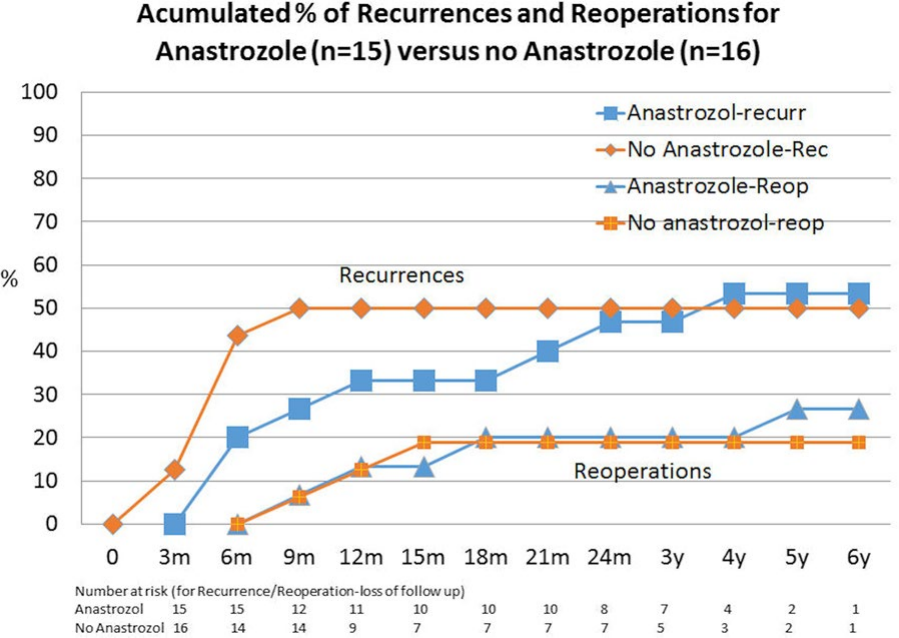
Accumulated % of recurrences and reoperations for Anastrozole versus no Anastrozole

Figure 4 shows the recurrence and reoperation rates observed during a 6-year follow-up period. Although the recurrence rate was similar at 2 years with or without Anastrozole (50%), the use of this AI delayed their appearance; however, differences were not statistically significant. In both groups with Mirena® + CS, there were few recurrences (29.4% at 3 months after treatment—AT—), with simple cysts or small endometriomas. However, in patients treated with Mirena® + TUGPA (groups 2 and 4), the endometriomas increased (together with reoperations) when the LNG-IUD was removed (see Table 2). Lines of the cumulative percentage of reoperations were also similar with or without Anastrozole.

*Fertility and clinical status* of patients in the last control are shown in Table 3. Ten percent of them got pregnant and 13% remained infertile. At 4.2 ± 1.7 years of follow-up (95%CI: 3.57–4.85; median 4 years, range 1–7 years), 25% of cases were reoperated, 13% showed persistent endometriosis (although these women evolved well taking pill or other medications—oral naproxen—), and 61.3% were asymptomatic without taking any medication. The more interesting finding is that 88% of the patients in which CS was performed, with or without Anastrozole, were asymptomatic after 3 to 5 years without medication or reoperation, compared with only 21% if TUGPA was performed, with or without Anastrozole. And these differences were significant between groups 1 and 2 (*p* = 0.004) both with Anastrozole and Mirena, and between groups 3 and 4 (*p* = 0.027) both with Mirena, being equally significant (*p* = 0.019) in the four groups.

**Table 3:** Fertility and clinical status in last control of the patients included in the clinical trial

Variable	Gr. 1. A + LNGIUD + CS [n = 8]	Gr. 2. A + LNGIUD + TUGPA [n = 7]	Gr. 3. LNGIUD + CS [n = 9]	Gr. 4. LNGIUD + TUGPA [n = 7]	Total CT [N = 31]
Years until last control	4.4 ± 1.8	5 ± 1.5	3.4 ± 1.3	4.2 ± 1.3	4.2 ± 1.7
Infertility	1 (12.5)	2 (28.6)	1 (11.1)	0–	4 (12.9)
Pregnancies/deliveries	0–	1 (14.3)^x^	1 (11.1)	1 (14.3)	3 (9.7)
*Clinical status in last control*					
1. Reoperated					
New CS	0	4 (57.1)*	0	3 (42.8)	7 (22.6)
Hyst + Adnexectomy	0–	0–	1 (11.1)	0–	1 (3.2)
2. Persist, well, taking OCP	1 (12.5)	2 (28.6)	0–	2 (28.6)	4 (12.9)
3. Well without medication	7 (87.5)*	1 (14.3)	8 (88.9)**	2 (28.6)	19 (61.3)***

Data are n(%) and mean ± SD. ^x^,1 case reoperation and then pregnancy. Statistical study.– H of Kruskal–Wallis: * between gr1 and gr2 *p*.004, ** between gr3 and gr4 *p*.027.—*** Chi-square Pearson among the 4 groups, *p*.019. A, anastrozole; CT, clinical trial; CS, conservative surgery; Hyst, hysterectomy

### Post-hoc or sensitivity analyses

No pathology related to the treatments was observed throughout the clinical trial follow-up period.

## Discussion

Our study shows that oral administration of 1 mg/day Anastrozole for 6 months, beginning before CS intervention of endometriosis, reduces or improves significantly the symptoms associated with the disease (especially dysmenorrhea and CPP) during and after treatment. No other significant advantages over the single insertion of LNG-IUD (Mirena®), prior to CS, were observed. The recurrence and reoperation rates were similar at 2 years with or without Anastrozole that were adversely influenced by the performance of TUGPA. These findings clarify what was previously reported about the use of Anastrozole in the treatment of endometriosis, suggesting that the clinical benefits reported after 6 months (pain relief, see Table S1) are partly due to the associated medications and that there are no other additional benefits about the endometriosis itself and its clinical evolution [3, 4, 12–15].

### Strengths and weaknesses of the study

The main strength of the study would be the strict randomization of cases of young women with endometriomas and elevated CA-125, for both patients taking or not Anastrozole and inclusion in CS or TUGPA during the medical treatment. A limitation of this research is the low number of cases included in the CT because of the low recruitment rate and the poor preliminary results observed in the interim in patients treated with TUGPA, which led us to stop recruitment. When the trial was planned, we trusted that TUGPA patients would evolve as well as those who were treated with CS, because of the additional use of Anastrozole and Mirena. However, the statistical tests are significant and, therefore, this is a valid CT that shows the poor results obtained performing TUGPA in endometriosis and which do not improve with the previous insertion of LNG-IUD (Mirena®) and/or oral Anastrozole for 6 months. A possible bias in this study could be the use of vitamin D (VD) in women taking Anastrozole, although the data on VD and endometriosis are controversial [16, 17].

### Discussion of the findings in relation to other studies

Initial studies and CTs had described the AIs as promising therapeutic agents for treatment of endometriosis [2, 4] since they could suppress the local estrogen produced by aromatase-positive implants and subsequent proliferative effect, as well as blocking the action on Cyclooxygenase-2 (COX-2) and prostaglandin E2, the later being responsible for inflammation [7, 18]. However, in most of these studies the use of AIs (Anastrozole or Letrozole) was associated with OCP or GnRH analogues, so its beneficial effects could correspond to these other medications or their association, rather than to the AIs themselves. Other case reports, series and prospective CTs using mainly Letrozole associated with noretindrone acetate (NETA) or OCP, compared to the use of the OCP or NETA alone [14, 15], have been published after 2008 (see Additional file 1: Table S1). Results do not show significant clinical advantages, but more cost and side effects related to the administration of Letrozole. Anastrozole was used only in 3 patients [19] with improvement of CPP and minimal side effects. Systematic reviews [20, 21] seem to conclude that AI may have a place in endometriosis treatment, but there is no clear evidence of improvement in endometriosis-associated infertility [22]; and the Committee Opinion No. 663 on Aromatase Inhibitors in gynecologic practice [23] pointed that AI are a promising therapeutic option that may be useful for the management of endometriosis-associated pain in combined therapy with progestins.

The pathogenesis of endometriosis remains controversial. A recent and extensive review on it [24] shows that the innate ability of endometrial stem cells to regenerate cyclically seems to play a key role in the development of endometriosis, but also the dysregulated hormonal pathways. A genetic dysregulation would cause aberrant placement of those stem cells, and then immune cells, adhesion molecules, extracellular matrix metalloproteinase, and pro-inflammatory cytokines activate / alter the peritoneal microenvironment, creating the conditions for differentiation, adhesion, proliferation and survival of ectopic endometrial cells [25]. But in addition, the intracellular production of estrogens in these cells probably can also favour the development of the disease. Aromatase P450 (enzyme that catalyzes the conversion of androgens into estrogens) has been found both in endometriotic tissue as in the eutopic endometrium of women with endometriosis [6, 26], and in endometriosis, there is also deficiency of 17β-hydroxysteroid dehydrogenase (17β-HSD2) (an enzyme that converts 17β-estradiol into estrone modulating exposure to the action of estrogens [27]. So that both the local production of estrogens and the loss of protective mechanisms determine a higher level of estradiol that characterizes both endometriosis and the eutopic endometrium of affected women [24]. Dislocated basal endometrial fragments in the peritoneal cavity can now induce chronic inflammation and tissue injury and repair (TIAR) mechanisms, which activate local estrogen production, proliferation, and infiltrative growth leading to endometriosis [28]. Later, the high concentration of estrogens and the overexpression of ERβ favor the survival and development of ER-positive ectopic tissue. Progesterone resistance is also a characteristic of endometriotic tissue compared to eutopic endometrium [29], and it is caused by the down-regulation of the progesterone receptor (PR) in ectopic tissue [30, 31]. Progesterone action is crucial to decreasing inflammation in the endometrium, and deviant progesterone signaling results in a proinflammatory phenotype which favours the establishment of ectopic endometrial implants.

In any case, when we proposed this prospective study to assess the efficacy of Anastrozole in endometriosis, we decided to associate it with the intrauterine insertion of an IUD containing LNG (for contraception and for a continued release of progestin). Previously Vercellini et al. [32] had published a pilot study using LNG-IUD versus expectant management after CS for symptomatic endometriosis with significant reduction of dysmenorrhea and risk of recurrence. Subsequently, other studies [9, 33–40] have also shown that LNG-IUD is an effective and well accepted treatment to reduce dyspareunia and dysmenorrhea and increase quality of life in women with suspected endometriosis. However, the long-term maintenance therapy using LNG-IUD after surgery was not effective for preventing endometrioma recurrence [38]. These evidences agree with our prospective CT that shows a greater improvement of the painful symptoms and a certain time delay in recurrence in patients taking Anastrozole (with Calcium + VD simultaneously), although there were no clear significant differences with the group of no Anastrozole or other positive efficacy data in the administration of Anastrozole associated with Mirena® (versus only Mirena®). The tumor marker levels were normalized more clearly in the patients who did not take Anastrozole and the rates of recurrence and reoperations were similar. The worst result in short and long term was the performance of TUGPA (instead of CS), independently of the use of Anastrozole and Mirena®.

Therefore, despite the fact that sometimes medical therapy (either Anastrozole/Mirena®, or Dienogest [41]) is sufficient to reduce the symptoms and signs of endometriosis, in a large number of patients it is necessary surgical eradication of lesions. In cases with intestinal deep infiltrating endometriosis, some authors [42, 43] propose its complete eradication with a nerve and vascular preserving approach to restore normal pelvic anatomy and its functions.

## Conclusions

Anastrozole for 6 months, beginning before CS of endometriosis, improves significantly the painful symptoms of endometriosis but it has no other significant advantages over the single insertion of LNG-IUD (Mirena®) during that same time period. LNG-IUD (Mirena®) associated with CS reduces significantly dysmenorrhea and dyspareunia, normalizes more effectively the values of CA-125, and also seems to decrease the rate of recurrences and long-term reoperations. However, Anastrozole / Mirena® associated with TUGPA are not very effective, so we deduce that these medications without surgery do not cure or significantly improve endometriosis itself.

Future research directions should be focused on the observation of subsequent fertility, as well as to the long-term recurrence in greater number of cases operated with CS after previous insertion of Mirena®, which could be maintained for 6–12 months, and even indefinitely, if patients do not want to get pregnant.

## Supplementary Information

**Additional file 1: Table S1.** Clinical publications on the use of Aromatase Inhibitors in women with endometriosis. CPP, chronic pelvic pain; VAS, visual analogue scale; OCP, oral contraceptive pill.

## Data Availability

The datasets used and/or analysed during the current stady available from the corresponding author on reasonable request.

## Abbreviations

AI: Aromatase inhibitors
CI: Confidence interval
CPP: Chronic pelvic pain
CS: Conservative surgery
CT: Clinical trial
GnRH: Gonadotropin-releasing hormone
LNG: Levonorgestrel
LNG-IUD: Levonorgestrel-releasing intrauterine device
OCP: Oral contraceptive pill
SD: Standard deviation
TUGPA: Transvaginal ultrasound-guided puncture-aspiration
TVU: Transvaginal ultrasound
VAS: Visual analogue scale
