# Supplementary material for: Anastrozole and levonorgrestrel-releasing intrauterine device in the treatment of endometriosis: a randomized clinical trial
Source: BMC Womens Health. 2021 May 20;21:211. doi: 10.1186/s12905-021-01347-9 (PMC8138989; doi:10.1186/s12905-021-01347-9)
Supplement: Supplementary file 1 — Additional file 1: Table S1. Clinical publications on the use of Aromatase Inhibitors in women with endometriosis. CPP, chronic pelvic pain; VAS, visual analogue scale; OCP, oral contraceptive pill. [file 12905_2021_1347_MOESM1_ESM.docx]

Table S1.- Clinical publications on the use of Aromatase Inhibitors in women with endometriosis. Ref, Reference; CPP, chronic pelvic pain; VAS, visual analogic scale; OCP, oral contraceptive pill.

| **Year** | **Author/Ref** | **Type of study** | **Indication** | **Medication** | **Months** | **N** | **Results at 6 months** |
| --- | --- | --- | --- | --- | --- | --- | --- |
| 1998 | Takayama et al.  Fertil Steril 1998;69:709-13. | Case report | Postmenopausal endometriosis that does not respond to medical or surgical treatments | Anastrozole | 9 | 1 | Pain relief. Endometriosis node reduction. |
| 2004 | Razzi et al. BJOG 2004;111:182-4 | Case report | Idem | Letrozole | 9 | 1 | Idem |
| 2004 | Ailawadi et al.  Fertil Steril 2004;81:290-6 | Pilot, prospective | Premenopausal endometriosis with unsatisfactory results in previous medical and surgical treatments. | Letrozole + Norethindrone acetate | 6 | 10 | Marked reduction of visible and histologically confirmed endometriosis in all 10 patients. 90% of pain reduction. |
| 2004 | Soysal et al.  Hum Reprod 2004;19:160-7 | Randomized | Premenopausal endometriosis | Anastrozole + GnRH analogs | 6 | 80 | 100% release of pain. |
| 2004 | Shippen et al.  Fertil Steril 2004;81:1395-8 | Case report | Premenopausal endometriosis that does not respond to medical and surgical treatments. | Letrozole + oral Progesterone 200 mg, rofecoxib, and calcitriol | 6 | 2 | Pain relief. Endometriosis node reduction. |
| 2005 | Fatemi et al.  Reprod Biomed Online 2005;11:455-7 | Case report | Agressive pelvic endometrioma in a post-menopausal patient. 10 year before: Hysterectomy and double adnexectomy | Letrozole | 18 | 1 | Eighteen months later, the endometrioma was almost completely regressed and patient was free of symptoms. |
| 2005 | Amsterdan et al.  Fertil Steril 2005;84;300-4 | Pilot, prospective | Premenopausal endometriosis that does not respond to medical and surgical treatments. | Anastrozole + OCP | 6 | 15 | 93% of pain relief |
| 2005 | Hefler et al.  Fertil Steril 2005;84:1033-6 | Pilot, not randomised | Recto-vaginal endometriosis | Anastrozol 0,25 mg transvaginal via | 6 | 10 | No significant changes, (low dose?) |
| 2007 | Mousa et al.  Obstet Gynecol 2007;109:1421-3 | Case report | Severe pelvic pain post-hysterectomy and double adnexectomy | Letrozol. No previous response to Exemestano |  | 1 |  |
| 2007 | Kimura et al.  Fertil Steril 2007;87:1468.e9-12 | Case report | Severe symptomatic adenomyosis | Anastrozole 1-2 mg/d and GnRH analogs | 4 | 1 | Reduction of uterine volume by 60% |
| 2007 | Remorgida et al.  Aust N Z J Obstet Gynaecol 2007;47:222-5 | Prospective, open-label | Endometriosis, stage IV | Letrozole + POP (Desogestrel) | 6 | 12 | Release of symptoms but cysts persist with subsequent recurrence. |
| 2007 | Remorgida et al.  Fertil Steril 2007;88:724-6 | Prospective, open-label | Rectovaginal endometriosis | Letrozole 2.5 mg/d + NETA 2,5 mg | 6 | 12 | Reduced the intensity of the symptoms but there was recurrence at 3 months |
| 2009 | Verma and Konje.  Eur J Obstet Gynecol Reprod Biol 2009;143:112-5 | Cases (4) | Premenopausal endometriosis-related CPP refractary to conventional treatment | Anastrozole (3 patients); Letrozole (1 patient) | 6 | 4 | Marked improvement in pelvic pain. Minimal side effects. |
| 2009 | Ferrero et al.  Hum Reprod 2009;24:3033-41 | Prospective, open-label, non-randomized trial | Pain symptoms caused by rectovaginal endometriosis | Combination of letrozole+noretisterone acetate or noretisterone acetate alone | 6 | 82 | Combination was more effective in reducing pain and deep dyspareunia but had more adverse effects, cost and no satisfaction. Similar recurrence of symptoms. |
| 2010 | Ferrero et al.  Eur J Obstet Gynecol Reprod Biol 2010;150-199-202 | Prospective pilot study | Colorectal endometriosis | Letrozole, 2.5 mg/day + Noretisterone acetate, 2.5 mg/day | 6 | 6 | Reduction of pain and gastrointestinal symptoms. |
| 2011 | Ferrero et al.  Gynecol Endocrinol 2011;27:337-40 | Cases (2) | Bladder endometriosis | Letrozole, 2.5 mg/day + Noretisterone acetate, 2.5 mg/day | 6 | 2 | Improve pain and urinary symptoms, but side effects and recurrence after interruption of treatment. |
| 2011 | Lall Seal et al.  Fertil Steril 2011;95:291.e15-8 | Cases (5) | Recurrence of endometriomas and CPP | Letrozole (2.5 mg/d)+ combined pill (desogestrel 0.15-EE 0.03) | 6 | 5 | Complete regression of cysts and pain relief in all cases. |
| 2011 | Alborzi et al.  Arch Gynecol Obstet 2011;284:105-10 | Prospective randomized clinical trial | Infertile women with endometriosis confirmed by prior laparoscopy | Group 1: Letrozole for 2 months. Group 2: Tripterelin for 2 months. Group 3: no medication. | 2 | 144( 47, 40 and 57 patients) | Pregnancy rate and endometriosis recurrence rate are comparable among the 3 groups. |
| 2014 | Almassinokiani et al.  Med J Islam Repub Iran 2014;28:107 | Prospective, randomized clinical trial | Pelvic endometriosis and pain score >5 (VAS) | Letrozole+OCP or only OCP | 4 | 51 (25 vs 26 patients) | Improvement of the VAS in both groups without differences between them. |
| 2014 | Ferrero et al.  Eur J Obstet Gynecol Reprod Biol 2014;174:117-22 | Patient-preference study | Endometriotic ovarian cysts | Noretisterone acetate (NETA) vs. Letrozole combined with NETA | 6 | 40(20 vs 20 patients) | Combination is more efficient in reducing the volume of endometriotic cysts but they did not disappear in any patient. |
